# Exposure to Night-Time Traffic Noise, Melatonin-Regulating Gene Variants and Change in Glycemia in Adults

**DOI:** 10.3390/ijerph14121492

**Published:** 2017-12-01

**Authors:** Ikenna C. Eze, Medea Imboden, Maria Foraster, Emmanuel Schaffner, Ashish Kumar, Danielle Vienneau, Harris Héritier, Franziska Rudzik, Laurie Thiesse, Reto Pieren, Arnold von Eckardstein, Christian Schindler, Mark Brink, Jean-Marc Wunderli, Christian Cajochen, Martin Röösli, Nicole Probst-Hensch

**Affiliations:** 1 Swiss Tropical and Public Health Institute, Socinstrasse 57, P.O. Box, CH-4002 Basel, Switzerland; medea.imboden@swisstph.ch (M.I.); maria.foraster@swisstph.ch (M.F.); emmanuel.schaffner@swisstph.ch (E.S.); ashish.kumar@unibas.ch (A.K.); danielle.vienneau@swisstph.ch (D.V.); harris.heritier@swisstph.ch (H.H.); christian.schindler@swisstph.ch (C.S.); martin.roosli@swisstph.ch (M.R.); nicole.probst@swisstph.ch (N.P.-H.); 2 University of Basel, Petersplatz 1, CH-4003 Basel, Switzerland; 3 Karolinska Institutet, SE-171 77 Stockholm, Sweden; 4 Center for Chronobiology, Psychiatric Hospital of the University of Basel, Wilhelm Klein-Strasse 27, 4056 Basel, Switzerland; franziska.rudzik@upkbs.ch (F.R.); laurie.thiesse@upkbs.ch (L.T.); christian.cajochen@upkbs.ch (C.C.); 5 Empa, Laboratory for Acoustics/Noise Control, Swiss Federal Laboratories for Material Science and Technology, Überlandstrasse 129, CH-8600 Dübendorf, Switzerland; reto.pieren@empa.ch (R.P.); jean-marc.Wunderli@empa.ch (J.-M.W.); 6 Institute of Clinical Chemistry, University Hospital Zurich, Rämistrasse 100, 8091 Zurich, Switzerland; arnold.voneckardstein@usz.ch; 7 Federal Office for the Environment, 3003 Bern, Switzerland; mark.brink@bafu.admin.ch

**Keywords:** transportation noise, *MTNR1B* gene, rs10830963, diabetes, glycosylated hemoglobin, circadian sleep-wake cycle, gene-environment interactions, adults, cohort study

## Abstract

Traffic noise has been linked to diabetes, with limited understanding of its mechanisms. We hypothesize that night-time road traffic noise (RTN) may impair glucose homeostasis through circadian rhythm disturbances. We prospectively investigated the relationship between residential night-time RTN and subsequent eight-year change in glycosylated hemoglobin (ΔHbA1c) in 3350 participants of the Swiss Cohort Study on Air Pollution and Lung and Heart Diseases in Adults (SAPALDIA), adjusting for diabetes risk factors and air pollution levels. Annual average RTN (Lnight) was assigned to participants in 2001 using validated Swiss noise models. HbA1c was measured in 2002 and 2011 using liquid chromatography. We applied mixed linear models to explore RTN–ΔHbA1c association and its modification by a genetic risk score of six common circadian-related *MTNR1B* variants (MGRS). A 10 dB difference in RTN was associated with a 0.02% (0.003–0.04%) increase in mean ΔHbA_1c_ in 2142 non-movers. RTN–ΔHbA1c association was modified by MGRS among diabetic participants (*P*_interaction_ = 0.001). A similar trend in non-diabetic participants was non-significant. Among the single variants, we observed strongest interactions with rs10830963, an acknowledged diabetes risk variant also implicated in melatonin profile dysregulation. Night-time RTN may impair glycemic control, especially in diabetic individuals, through circadian rhythm disturbances. Experimental sleep studies are needed to test whether noise control may help individuals to attain optimal glycemic levels.

## 1. Introduction

Glycosylated hemoglobin (HbA1c) reflects three-month average glycemia [1], predicts diabetes in non-diabetic individuals, and complications in diabetic individuals [2,3]. Despite control efforts, the prevalence of poorly-controlled diabetes remains high [4]. Recent interest in the role of environmental stressors in cardio-metabolic diseases towards improved prevention [5] makes it pertinent to explore the environmental determinants of glycemic control. Hyperglycemia is an important risk factor for cardiovascular diseases [6,7,8] and diabetes [2,3]. Understanding the role of environmental stressors also entails investigations of their mediating or modifying mechanisms, in persons with and without diabetes.

Road traffic noise (RTN) represents the most common transportation noise, and together with air pollution, constitutes more than 75% of environmental disease burden in Europe [9]. Growing evidence suggests a link between RTN and diabetes morbidity [10,11,12,13,14] and mortality [15] (independent of traffic-related air pollution), with unclear mechanisms [5,16].

**Figure 1:**
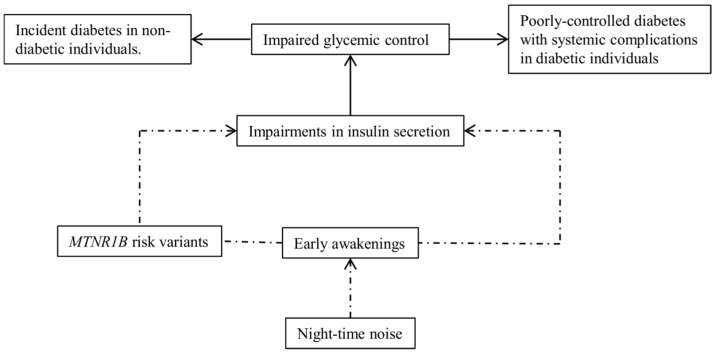
Hypothesized mechanisms of noise effects on glucose homeostasis explored in the present study.

Melatonin, a pineal hormone known to act on numerous organs and co-regulate various neural and endocrine processes, exhibits a circadian rhythm which is closely linked to sleep propensity [17] and insulin secretion [18]. Administration of exogenous melatonin elicited subjective sleepiness [19] and glucose intolerance when given close to mealtime [20]. Melatonin mediates its regulatory function through binding to receptors including the high-affinity melatonin receptor 1B encoded by *MTNR1B*, a gene widely expressed in the human retina and pancreatic islet cells. Recent genome-wide association studies (GWAS) showed seven common variants near *MTNR1B* to be associated with glucose homeostasis or type 2 diabetes, mainly through impairments in insulin secretion [21]. The glucose-raising allele of the lead variant, rs10830963, was shown to significantly prolong the duration of elevated melatonin levels by 41 min, and delayed the circadian phase of dimlight melatonin offset by 1.37 h, as well as increased diabetes risk among individuals who woke up early [22]. Individuals who are evening chronotypes were also shown to have higher risk of diabetes [23]. In both instances, it is thought that waking up early or sleeping late (during high melatonin and low insulin phase) predisposes to having meals, leads to high insulin levels and subsequent hyperglycemia, as a result of altered timing of food intake. Thus, the glucose-raising allele of rs10830963 could modify susceptibility to noise exposure in cases of early awakenings during melatonin secretion (Figure 1).

Night-time noise exposure could impair glucose homeostasis through disturbances in circadian sleep–wake cycles. Circadian pathway was captured using melatonin-regulating genetic variants as a proxy, possibly reflecting genetic risk for melatonin profile dysregulation.

Although there are at least seven common variants on *MTNR1B* influencing glucose metabolism through a common pathway [21], sleep and circadian studies have only focused on rs10830963 variant [24]. In essence, following the approach of using scores involving multiple variants for improved prediction of genetic risks [25,26], an *MTNR1B* genetic risk score (MGRS) based on variants that are not in strong linkage disequilibrium (LD), may better capture individuals at high-risk of melatonin profile dysregulation than single variants.

Understanding the implications of noise exposure on glucose homeostasis through interactions with circadian-related parameters will improve our understanding and offer preventive channels towards optimal glucose control in diabetic and non-diabetic individuals.

We therefore tested the hypothesis that MGRS (and component single variants representing the circadian pathway) may modify the potential association between night-time RTN and eight-year change in HbA1c (ΔHbA1c) in diabetic and non-diabetic participants of the Swiss Cohort Study on Air Pollution and Lung and Heart Diseases in Adults (SAPALDIA).

## 2. Materials and Methods

### 2.1. Study Population

Participants included 3350 adults aged 29–81 years who completed health interviews and examinations at the first (SAP2) and second (SAP3) SAPALDIA follow-up surveys, and had complete information on relevant covariates. Details of inclusion are shown in Figure S1. The SAPALDIA study began in 1991 (SAP1), with 9651 randomly-selected adults from eight geographically-representative Swiss areas [27]. Two follow-up surveys in 2001/2002 and 2010/2011 retained 8047 and 6088 participants, respectively. At each survey, participants responded to detailed questions concerning their health and lifestyle. At SAP2 and SAP3, blood was sampled into a biobank for biomarker assays, including HbA1c and genotyping. Participants provided written informed consent and ethical clearances were obtained from the Ethics Committees of the Swiss Academy of Medical Sciences and the participating cantons (National Ethics Committee for Clinical Research (UREK); Project Approval Number 123/00; date of approval: September 2001).

### 2.2. Measurement of HbA1c and Identification of Diabetes Cases

HbA1c was measured in EDTA-buffered whole blood samples collected at SAP2 and SAP3 using the ARK-RAY ADAMS A1c HA-8180V Analyser (Menarini, Florence, Italy) based on high-performance liquid chromatography, which has minimal interference from alternate hemoglobin variants [28]. HbA1c values were measured in mmol/mol, and converted into percentage [29]. Using a combination of questionnaire data and HbA1c values, we identified participants as diabetes cases if they (I) self-reported physician-diagnosed diabetes, (II) used diabetes medication, or (III) had a HbA1c value ≥6.5% at SAP2 or SAP3. We also defined confirmed/advanced diabetes, restricted to only participants taking medication, thus, yielding three comparative groups: no diabetes (at SAP2 and SAP3), diabetes (at SAP2 or SAP3), and diabetes on medication (at SAP2 or SAP3). We defined ΔHbA1c as the absolute difference between HbA1c at SAP3 (2011) and HbA1c at SAP2 (2002).

### 2.3. Exposures

Average annual exposure to RTN was assigned to participants based on the most exposed façade of their residential floors in 2001 and 2011 (corresponding to the survey time points) using the SonROAD emission and STL-86 propagation models combining high-resolution spatial and temporal road traffic information, as previously described [30]. This was done in the framework of the SiRENE (Short and Long-Term Effects of Transportation Noise Exposure) project, where railway and aircraft noise were also assigned to the same façade using sonRAIL propagation and SEMIBEL emission models for railway noise, and FLULA2 simulation model for aircraft noise—all validated Swiss noise models [30]. Day (Lday; 07–23 h), night (Lnight; 23–07 h), and day–evening–night (Lden) noise (dB) were computed for road, railway, and aircraft sources. Participants without substantial night-time railway/aircraft exposures were assigned truncated values of 20 dB, and were assigned a truncation indicator. Lnight was highly correlated with constituent time points (23–01 h, 01–05 h and 05–07 h; Spearman R > 0.9). Day and night-time correlations (Spearman R) of road traffic, aircraft, and railway noise were 0.99, 0.33, and 0.93, respectively. Since our study was aimed at modification of noise–HbA1c association by circadian-related parameters, and RTN is the most common noise with consistently reported associations with diabetes mortality/morbidity [12], we focused our analyses on night-time RTN, considering night-time railway and aircraft noise as potential confounders, with secondary exploration of their main associations. Noise levels were quite stable over follow-up (Spearman R > 0.9 for both time points), thus, the noise levels at 2001 would capture long-term exposures in statistical models predicting health outcomes after 2001. To limit exposure misclassification to an extent, we also restricted analyses to SAPALDIA participants who did not change their residence during the follow-up period.

### 2.4. Potential Confounders

From the questionnaire data at SAP2, we extracted additional potential confounders of the association between RTN at SAP2 and change in HbA1c between SAP2 and SAP3. These included age (continuous), sex (male/female), formal education (≤9 (primary level)/10–13 (secondary, apprenticeship level)/>13 years (tertiary level)), neighborhood socio-economic index (SEI) (continuous; incorporating education and income of household head, median rent and crowding of households within the neighborhood) [31], and green areas within 2 km residential buffer, available from the European Environment Agency hectare resolution dataset (continuous; CORINE CLC-2006 Version 13). We also considered smoking status (never/former/current), exposure to passive smoke (yes/no), consumption of alcohol (including liquor, wines, beers and spirits; ≤1/>1 glass/day), and body mass index (BMI), defined as weight and height-squared ratio (continuous). Towards sensitivity analyses, we extracted participants’ responses to the question “do you often feel that you have not slept enough after you wake up in the morning?” from which we derived insufficient sleep (yes/no), as well as their responses to the Short Form 36-item mental health questionnaire (continuous) [32]. In SAP2, average annual residential exposure to nitrogen dioxide (NO_2_), a marker of road traffic-related air pollution and potential confounder of RTN [33], was assigned to participants’ residences based on a hybrid model (adjusted R^2^ = 0.83) derived by fitting NO_2_ sampler measurements using land-use and traffic variables, and predictions from a Gaussian dispersion model as previously described [34].

### 2.5. MTNR1B Gene Score

Genomic DNA was extracted from EDTA-buffered whole blood using Puragene™ DNA Isolation Kit (Gentra Systems, Plymouth, UK). Genotyping of SAPALDIA participants was done on DNA samples collected at SAP2. In the framework of the EU-funded GABRIEL consortium to identify genetic determinants of asthma [35], 1612 participants were genotyped using Illumina Human 610Kquad BeadChip (G1; Illumina, San Diego, CA, USA) covering ~570,000 variants. An additional 3015 participants were genotyped using Illumina Human OmniExpress-Exome BeadChip (G2; Illumina, San Diego, CA, USA), covering ~1 million variants. Quality control criteria were applied to both genotyping arrays: samples with <97% genotyping success rate, or of non-European origin, with cryptic relatedness or sex inconsistencies, were excluded. Variants with minor allele frequency (MAF) of <5% or deviation from Hardy–Weinberg equilibrium (HWE) at a threshold of 10^−6^ were also excluded. G1 and G2 datasets were phased using ShapeIT version 2.r790 [36] and imputed using MiniMac2 [37]. The imputed datasets were then merged, after excluding variants with low imputation quality (R^2^ < 0.3), yielding ~14 million markers for 4324 participants across G1 and G2, from where we identified seven common *MTNR1B* variants (rs1387153, rs10830962, rs4753426, rs8192552, rs10830963, rs3781638 and rs2166706) involved in glucose dysregulation [21]. Imputations were of high quality. All but rs10830963 (R^2^ ≥ 0.87) had an imputation R^2^ ≥ 0.92. All variants had similar allele frequencies in comparison with other studies [38,39,40,41,42,43,44,45,46,47,48,49] and the 1000 Genomes Central European Population [50]. All variants were in HWE (*p* > 0.2), with MAF ≥ 7% (Table S1). Two variants, rs10830962 and rs2166706, were in high LD (R^2^ = 0.89; Figure S2), thus, we excluded rs2166706 from the analyses. The LD R^2^ of the included MTNR1B variants ranged between 0.2 and 0.7. Since the glucose-raising allele of rs10830963 was the allele implicated in melatonin profile dysregulation, expressed as a significant delay in melatonin offset [22], we coded the other variants in their reported direction of association in glucose alterations, such that each variant contained 0–2 quantities of the risk allele. We created MGRS by summing up risk alleles across the six variants, yielding a minimum, median, and maximum of 2, 6, and 12 risk alleles, respectively. We also created a categorical variable—low risk (MGRS ≤ 6) and high risk (>6).

### 2.6. Statistical Analyses

We summarized the characteristics of participants by inclusion and exclusion status. Differences in proportions and means were tested using chi-squared and *t*-tests respectively. We built predictive statistical models using noise exposure and covariates measured at SAP2, for ΔHbA1c. Both crude and adjusted associations between MGRS (as well as the single variants, in separate models) and ΔHbA1c; and between RTN and ΔHbA1c were assessed using mixed linear models with a random intercept at the level of study area. Adjusted models included age, sex, education, neighborhood SEI, smoking status, alcohol consumption, and BMI. Adjusted RTN–ΔHbA1c models additionally included NO_2_, railway and aircraft noise, noise truncation indicators, and green space within a 2 km residential buffer. We included interaction terms between RTN and MGRS (as well as the single variants, in separate models), in the RTN–ΔHbA1c models, to explore the presence of interactions between these variables. We also stratified the RTN–ΔHbA1c model by categories of MGRS. Furthermore, we restricted analyses to participants who did not change their residence during follow-up. Since our study included only 35% of SAP1 participants, we limited potential selection bias by applying the inverse of the probability of participating in present analyses as weights in our models. These probability weights were derived from a logistic regression model using predictor variables from SAP1. We performed sensitivity analyses: we explored cross-sectional associations between MGRS (as well as the single variants, in separate models) and HbA1c using repeated mixed linear models with random intercepts at the level of participants. Using the RTN–ΔHbA1c model, we tested sensitivity to removal of BMI from the main and interaction models, performed complete case analyses without adjusting for potential selection bias, and excluded asthmatic participants in the RTN–MGRS interaction model to explore potential genotyping selection bias. We explored interactions with self-reported sleep insufficiency reported at SAP2. All analyses were stratified into three categories—no diabetes, diabetes, and diabetes on medication—to limit confounding/effect modification by diabetes status or medication. All analyses were done using STATA version 14 (STATA Corporation, College Station, TX, USA). Statistical significance was defined at two-sided alpha-values of 0.05 and 0.1 for main associations and interactions, respectively.

## 3. Results

### 3.1. Characteristics of Participants

Over an approximate eight-year follow-up, the mean increase in HbA1c was 0.04%, whereas diabetes prevalence was 3% at SAP2 and 7% at SAP3, with 8% combined prevalence in the 3350 included participants. On average, diabetic participants gained more HbA1c than non-diabetic participants (Table 1), in line with evidence for poor glucose control in Switzerland [51]. 

SAP2 and SAP3 represent the first and second follow-up surveys of the Swiss Cohort Study on Air Pollution and Lung and Heart Diseases in Adults in 2002 and 2010/2011, respectively. Lnight represents night-time (23–07 h) noise levels assigned to the SAPALDIA participants based on the most exposed façade of their residential floors. Number of included participants is 3350.

Mean (SD) annual exposures to night-time road traffic, railway, and aircraft noise at SAP2 were 46 (8) dB, 28 (10) dB, and 23 (6) dB, respectively. Figure S3 shows the distribution of noise exposures in the included participants. Mean (SD) MGRS was 6 (3) risk alleles. Compared to the participants excluded due to non-participation during follow-up or missing data, included participants were younger, more likely males, better educated, and less likely to be overweight or diabetic. They also had lower exposure to RTN and NO_2_, but higher exposure to aircraft noise. There were no significant differences in ΔHbA1c, MGRS, and change of residence between both groups (Table S2).

### 3.2. Associations between RTN and ΔHbA1c

In non-diabetics, we observed generally non-significant associations between transportation noise and ΔHbA1c. Among diabetics, associations with railway and aircraft noise were positive (reaching significance only for aircraft noise), whereas associations with road traffic noise were negative. Limiting analyses to non-movers revealed consistent positive associations between RTN and ΔHbA1c among both diabetic and non-diabetic participants. Among non-diabetic non-movers, mean ΔHbA1c showed a statistically significant increase by 0.01% (95% CI 0, 0.03) per 10 dB exposure to RTN. Among diabetic non-movers, associations were reversed compared to all participants, became positive, and were stronger than we observed in non-diabetic participants. Associations also reached ten-fold in those reporting the use of diabetic medication compared to non-diabetics, but were not significant (Table 2). 

All estimates represent increase (+) or decrease (−) in mean change in HbA1c per 10 dB of night-time road traffic noise. Adjusted models included age, sex, education, neighborhood socio-economic index, smoking status, passive smoking, alcohol consumption, green space within a 2 km residential buffer, residential levels of nitrogen dioxide, night-time railway, aircraft noise and their truncation indicators. All models include random intercepts at the level of the study areas, and were adjusted for potential selection bias by applying the probability of participation in present analyses as weights derived from a logistic regression with predictors from the baseline study in 1991.

In the adjusted models, we also found significantly positive associations of ΔHbA1c with aircraft noise in non-diabetics, where mean HbA1c increased by 0.02% (95% CI 0, 0.03) per 10 dB difference in Lnight. There were no significant associations with railway noise. Similar to RTN, associations of ΔHbA1c with aircraft noise in non-movers were consistently positive across comparison groups (Table 2). All models were stable to confounder adjustments, including BMI. Irrespective of subpopulation studied or confounder adjustments, we did not observe any association of railway noise with ΔHbA1c. 

### 3.3. Associations between MGRS and ΔHbA1c

Results of main associations between MGRS (and component variants) and HbA1c are presented in Table S4. In non-diabetic participants, MGRS showed a positive association with ΔHbA1c and cross-sectional HbA1c, but only reached significance in the cross-sectional analysis. All six single variants were positively associated with HbA1c, with associations of 0.01–0.03% per risk allele. Among diabetic participants, there was a non-significant tendency for associations of MGRS (and component variants) with ΔHbA1c and cross-sectional HbA1c to be negative.

### 3.4. Modification of RTN-ΔHbA1c Association by MGRS

**Figure 2:**
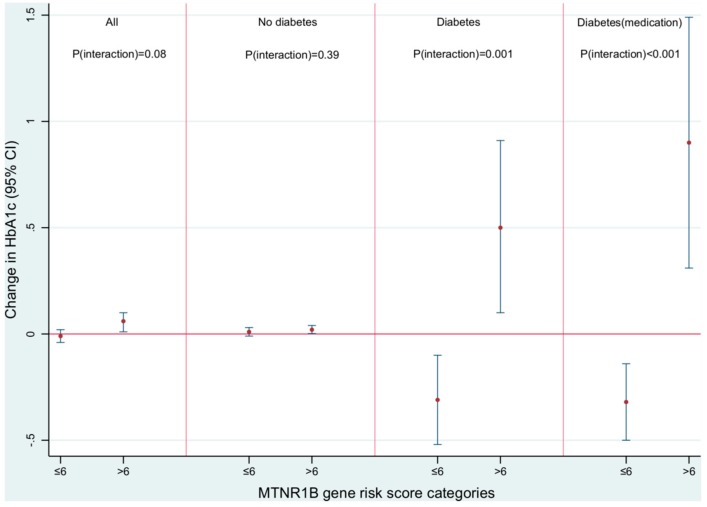
Modification of the association between night-time road traffic noise and change in HbA1c by melatonin dysregulation risk score, in non-movers.

Among non-movers, we observed significant interactions between MGRS and RTN that were restricted to diabetic participants. The interactions were stronger in persons who reported medication use where mean ΔHbA1c changed by 0.90% (0.31, 1.49%) in diabetic participants on medication with high MGRS, and by −0.32% (−0.50, −0.14%) per 10 dB, in those with low MGRS (*P*_interaction_ = 0.001) (Figure 2). All single variants showed positive interaction with RTN in diabetic participants. The lead functional variant, rs10830963, showed the strongest significant interactions, where mean ΔHbA1c increased by 0.80% (0.14, 1.47%) and by 1.21% (0.59, 1.83%) per 10 dB and per risk allele, in diabetics and medicated diabetics, respectively (Table 3).

Interaction terms included night-time road traffic noise and *MTNR1B* variants/score. *MTNR1B* score represents the sum of the risk alleles across six included variants. Positive sign of beta coefficient means increase in HbA1c per 10 dB change in night-time road traffic noise and per risk allele. All models were adjusted for age, sex, education, neighborhood socio-economic index, smoking status, passive smoking, alcohol consumption, green space within a 2 km residential buffer, residential levels of nitrogen dioxide, night-time railway, aircraft noise, and their truncation indicators. All models included random intercepts at the level of the study areas, and were corrected for potential selection bias by applying the probability of participation in present analyses as weights derived from a logistic regression with predictors from the baseline study in 1991.

Although we observed statistically significant associations between RTN and mean ΔHbA1c among non-diabetic participants with high MGRS, the difference in associations between non-diabetic participants with high and low MGRS was non-significant (*P*_interaction_ = 0.39) (Figure 2).

All results are presented as increase or decrease in mean change in HbA1c per 10 dB of night-time road traffic noise. *MTNR1B* genetic risk score represents the sum of risk alleles across six included *MTNR1B* variants. All models were adjusted for age, sex, education, neighborhood socio-economic index, smoking status, passive smoking, alcohol consumption, body mass index, green space within a 2 km residential buffer, residential levels of nitrogen dioxide, night-time railway, aircraft noise and their truncation indicators. All models include random intercepts at the level of the study areas, and were corrected for potential selection bias by applying the probability of participation in present analyses as weights derived from a logistic regression with predictors from the baseline study in 1991.

The interactions between RTN and MGRS did not significantly differ by sex in both diabetic and non-diabetic participants (*P*_interaction_ ≥ 0.30). Similar to the main models, interaction models were also very stable to adjustments for BMI (Table 3). Even though the direction of interaction terms in models including all participants was generally similar to those in non-movers, the magnitude of interactions was smaller, and the interaction terms in those models were not statistically significant (Table S3). 

### 3.5. Sensitivity Analyses

Our results were robust to sensitivity analyses, including complete case analyses without correction for potential selection bias, as well as RTN–MGRS interaction analyses excluding asthmatic participants Sensitivity analyses limited to participants whose bedrooms were oriented towards the street also showed very similar results. Interactions with self-reported sleep insufficiency were consistent in diabetic participants, reaching significance among those on medication. Unlike with MGRS, we observed significant interaction with self-reported sleep sufficiency in non-diabetic individuals (Table 4).

*MTNR1B* score represents the sum of risk alleles across six included variants. All estimates represent an increase (+) or decrease (−) in mean change in HbA1c per 10 dB of night-time road traffic noise. Adjusted models included age, sex, education, neighborhood socio-economic index, smoking status, passive smoking, alcohol consumption, body mass index, green space within a 2 km residential buffer, residential levels of nitrogen dioxide, night-time railway, aircraft noise, and their truncation indicators. All models include random intercepts at the level of the study areas, and were adjusted for potential selection bias by applying the probability of participation in present analyses as weights derived from a logistic regression with predictors from the baseline study in 1991. NA: not applicable.

## 4. Discussion

We found positive associations between exposure to RTN and eight-year change in HbA1c in non-movers, which were significantly stronger among diabetic individuals at genetic risk of circadian rhythm disturbances. We also found positive associations with aircraft noise that were again stronger in diabetics. Railway noise was not associated with change in HbA1c, supporting findings from previous studies where road traffic and aircraft noise, but not railway noise [12,13] was associated with diabetes risk.

Melatonin is involved in the regulation of human circadian rhythms through its role in thermoregulation and sleep induction [17]. The secretion of melatonin occurs during the biological night, in a fasting state, when insulin secretion is low [52]. Therefore, the melatonin pathway may play a role in noise susceptibility if noise exposure causes early awakenings and potential early meals, which could stimulate insulin secretion during high melatonin levels, leading to impaired glucose tolerance [22,53]. Diabetics with high MGRS had higher mean BMI (31 kg/m^2^) compared to those with low MGRS (29 kg/m^2^). Noise could also delay sleep onset [54], possibly leading to later chronotype which was associated with metabolic disturbances and diabetes [23,55]. We found supportive evidence for this hypothesis only in diabetic participants. One explanation for this may be that individuals with diabetes usually have comorbidities and worse homeostatic mechanisms, and are more prone to environmental stressors [56,57]. Exposure to high traffic volume was shown to have a stronger impact among diabetic individuals on insulin, which could imply more complicated diabetes, expressing higher inflammatory profiles compared to those on oral hypoglycemic agents [58]. Participants using diabetic medication in this study were more likely to be overweight, to have cardiovascular diseases and higher C-reactive protein levels compared to the other participants. Diabetic individuals with a high genetic risk for melatonin profile dysregulation may be particularly sensitive to poor glucose homeostasis. Diabetic individuals have also been shown to have sleep–wake cycle irregularities compared to non-diabetics [59,60]. Our finding of a significant negative effect of noise on glycemia among diabetics with low MGRS is surprising. Although this could be a chance finding, diabetics with low MGRS had lower average night-time noise exposure (46 dB), and were less noise sensitive (45%) compared to those with high MGRS (48 dB noise level and 51% noise sensitivity). Diabetic participants with low MGRS potentially have better melatonin profiles, and could be at lower risk for noise disturbances. The complexity of the melatonin system in influencing several physiological processes [24] calls for more research to better understand this finding.

Our observation of interaction of RTN with self-reported sleep insufficiency, but not with genetic risk for melatonin profile dysregulation on ΔHbA_1c_ in non-diabetic individuals, suggests that the melatonin pathway may not be relevant in this group. Interestingly, noise-induced sleep disruption was reported to impair glucose homeostasis through non-melatonin pathways, including the activation of sympathetic nervous system and release of stress-related hormones [61]. As GWAS continues to identify more circadian-related variants, future studies should consider variants covering the entire circadian pathways, and also incorporate objective sleep and stress-related parameters to improve understanding of the cardiometabolic effects of noise.

Since night-time noise levels were correlated to Lden, we may not exclude the contributions of day-time noise exposure (potentially via the stress pathway) to our observations. Noise, through stress/anxiety, may reduce adherence to medication in diabetic individuals, worsening their glucose control [62,63]. Exploratory analyses among the respondents to the SF-36 mental health survey [32] showed a reduction in the magnitude of the observed interactions (in diabetic participants) following adjustment for their mental health scores (Table 3).

The strengths of this study derive from its novelty in applying gene–environment interactions to better understand the impact of noise on glucose control in a longitudinal design, and the availability of detailed phenotypic and genotypic information in the SAPALDIA study. Information on medication use allowed the exploration of different diabetes phenotypes. We could test the hypothesis covering a potential pathway of glycemic effects of noise exposure, and used validated models with high spatial resolutions to assign individual estimates of noise and air pollution. The availability of information on change of residence allowed focusing analyses on non-movers, allowing the use of baseline noise levels as long-term exposures towards our health outcome.

Although our study is limited by sample size which calls for cautionary interpretation, we controlled for potential selection bias and made salient findings which could be generalized to all non-movers in the study period. Potentially, post-transcriptional/translational modifications may have affected melatonin profile, hence the absence of a risk variant may not imply normal melatonin profiles, and vice versa. We also lack information on melatonin drug use by the participants. However, the lack of correlation (R = 0.01) between self-reported sleep insufficiency and MGRS in our study corroborates the findings of previous studies where the lead *MTNR1B* variant was not associated with sleep duration [22,64], validating our application of MGRS in this study. We did not have adequate nutrient intake information e.g., antioxidant or fiber intake. Adjustment for nutrients may be considered an over-adjustment, if part of the noise–HbA1c association is mediated by impact of noise on food preference. Although our noise estimates were from validated models, some degree of misclassification will have occurred due to errors in input data. We did not have information on participants’ work shifts in our noise exposure models. However, the resulting bias is more likely non-differential, and to bias effect estimates towards null. We did not consider window opening habits which may be related to exposure level, and could have also biased our estimates towards null. Since we found significant associations in non-movers, we cannot generalize our findings to all SAPALDIA participants. Noise exposure metrics were significantly different between movers and non-movers, with a tendency for movers to move to areas with lower RTN (Table S6). This tendency per se, might have led to an over-estimation of the noise effects among movers if there had been no random misclassification. However, the total bias is the sum of any bias associated with differential misclassification, and the attenuation bias associated with random misclassification. Although we cannot prove it, we are quite convinced that the attenuation bias had a stronger impact on our results than any potential bias due to differential misclassification.

## 5. Conclusions

Our findings raise the hypothesis that genetic risk for melatonin profile dysregulation, in combination with long-term road traffic noise, better captured in non-movers, may increase the risk for poor glucose homeostasis, particularly in diabetic patients. While our findings need replication and confirmation in other independent cohorts, experimental interventional studies should test the hypothesis to determine if counseling for noise control should be added to diabetes care.

## Figures and Tables

**Table 1:** Characteristics of study participants included in the study.

**Categorical Variables**	**(%)**
Females at SAP2	48
Formal education at SAP2: ≤9 years	4
10–13 years	64
>13 years	32
Ever-smoker at SAP2	55
Exposure to passive smoke at SAP2	23
Alcohol consumption >once/day at SAP2	40
Sleep insufficiency at SAP2	28
Diabetes status at SAP2	3
Diabetes status at SAP3	7
Diabetes medication at SAP2	3
Diabetes medication at SAP3	5
Change of residence (movers) between SAP2 and SAP3	36
**Continuous Variables (Units)**	**Mean (SD)**
Age at SAP2 (years)	51 (11)
Body mass index at SAP2 (kg/m^2^)	25.6 (4)
Neighborhood socio-economic index at SAP2 (%)	64 (10)
SF-36 mental health score at SAP2 (%) ^a^	76 (15)
Lnight, road at SAP2 (dB)	45.8 (8)
Lnight, railway at SAP2 (dB)	28.3 (10)
Lnight, aircraft at SAP2 (dB)	22.7 (6)
Nitrogen dioxide at SAP2 (μg/m^3^)	22.4 (10)
Green space within 2 km radius at SAP2 (km^2^)	0.3 (0.4)
*MTNR1B* variants at SAP2: rs1387153, T allele	0.6 (0.6)
rs10830962, G allele	0.8 (0.7)
rs4753426, C allele	1.0 (0.7)
rs8192552, G allele	1.9 (0.4)
rs10830963, G allele	0.6 (0.6)
rs37816938, G allele	1.1 (0.7)
*MTNR1B* genetic risk score at SAP2	6 (3)
Glycosylated hemoglobin, HbA1c at SAP2 (%)	5.2 (0.4)
HbA1c at SAP3 (%)	5.3 (0.5)
Change in HbA1c between SAP2 and SAP3 (%): All	0.04 (0.4)
Non-diabetic participants	0.03 (0.3)
Diabetic participants	0.17 (1.1)
Diabetic participants on medication	0.12 (1.2)

^a^ N (included) = 2933.

**Table 2:** Exposure to night-time transportation noise in 2001 (10 dB difference) and subsequent change in HbA1c (%).

**All Participants**	**Source**	**Model**	**All** **N = 3350**	**No Diabetes** **N = 3098**	**Diabetes** **N = 251**	**Diabetes on Medication** **N = 168**
			**β (95% CI)**	**β (95% CI)**	**β (95% CI)**	**β (95% CI)**
	Road	Crude	0.01 (−0.01, 0.03)	0.01 (0.002, 0.02) *	−0.02 (−0.22, 0.18)	−0.03 (−0.25, 0.18)
		Adjusted	0.01 (−0.02, 0.03)	0.01 (−0.0004, 0.02) †	−0.07 (−0.26, 0.11)	−0.13 (−0.36, 0.10)
		Adjusted+BMI	0.01 (−0.02, 0.03)	0.01 (−0.001, 0.02) †	−0.08 (−0.27, 0.11)	−0.11 (−0.35, 0.15)
	Railway	Crude	0.004 (−0.01, 0.02)	0.003 (−0.01, 0.01)	0.03 (−0.06, 0.12)	0.03 (−0.11, 0.16)
		Adjusted	0.001 (−0.01, 0.02)	0.001 (−0.01, 0.01)	0.01 (−0.05, 0.08)	0.05 (−0.05, 0.13)
		Adjusted+BMI	0.002 (−0.01, 0.02)	0.001 (−0.01, 0.01)	0.02 (−0.05, 0.08)	0.04 (−0.04, 0.12)
	Aircraft	Crude	−0.0003 (−0.01, 0.01)	−0.01 (−0.03, 0.01)	0.24 (−0.08, 0.55)	0.26 (0.05, 0.47) *
		Adjusted	0.005 (−0.02, 0.03)	−0.004 (−0.03, 0.02)	0.34 (0.12, 0.55) †	0.34 (0.13, 0.54) †
		Adjusted+BMI	0.01 (−0.01, 0.02)	−0.003 (−0.02, 0.02)	0.35 (0.08, 0.62) †	0.31 (0.03, 0.60) †
**Non-movers**	**Source**	**Model**	**All** **N = 2142**	**No Diabetes** **N = 1960**	**Diabetes** **N = 179**	**Diabetes on Medication** **N = 117**
	Road	Crude	0.02 (0, 0.04) *	0.01 (0.0004, 0.03) *	0.06 (−0.21, 0.33)	0.07 (−0.26, 0.39)
		Adjusted	0.02 (0.003, 0.04) *	0.01 (0, 0.03) *	0.04 (−0.16, 0.24)	0.10 (−0.13, 0.33)
		Adjusted+BMI	0.02 (0.003, 0.04) *	0.01 (0, 0.03) *	0.03 (−0.14, 0.19)	0.10 (−0.09, 0.29)
	Railway	Crude	0.001 (−0.03, 0.03)	−0.01 (−0.02, 0.01)	0.06 (−0.15, 0.26)	0.05 (−0.31, 0.41)
		Adjusted	−0.001 (−0.03, 0.03)	−0.01 (−0.02, 0.01)	0.02 (−0.29, 0.31)	−0.03 (−0.53, 0.47)
		Adjusted+BMI	−0.001 (−0.03, 0.03)	−0.01 (−0.02, 0.01)	0.02 (−0.24, 0.29)	−0.03 (−0.46, 0.40)
	Aircraft	Crude	0.03 (0.01, 0.05)	0.01 (−0.01, 0.03)	0.24 (−0.04, 0.53) †	0.22 (−0.03, 0.47) †
		Adjusted	0.04 (0.01, 0.07) †	0.02 (−0.002, 0.03) †	0.30 (−0.15, 0.74)	0.14 (−0.44, 0.71)
		Adjusted+BMI	0.04 (0.01, 0.07) †	0.02 (0, 0.03) *	0.32 (−0.22, 0.86)	0.13 (−0.49, 0.74)

* *p* < 0.05; † *p* < 0.1.

**Table 3:** Interaction between *MTNR1B* variants (single/score) and night-time road traffic noise (10 dB difference) on change in HbA1c, in non-movers.

*MTNR1B* Variant	Risk/Other Allele	Risk Allele Frequency	All N = 2142	No Diabetes N = 1960	Diabetes N = 179	Diabetes on Medication N = 117
			β Interaction Term (95% CI)	β Interaction Term (95% CI)	β Interaction Term (95% CI)	β Interaction Term (95% CI)
rs1387153 ^a^	T/C	0.30	0.04 (−0.02, 0.10)	0.01 (−0.01, 0.02)	0.66 (0.17, 1.15) *	0.92 (0.45, 1.39) †
rs1387153 ^b^	T/C	0.30	0.04 (−0.02, 0.10)	0.01 (−0.01, 0.02)	0.67 (0.17, 1.18) *	0.92 (0.44, 1.40) †
rs10830962 ^a^	G/C	0.42	0.03 (−0.02, 0.09)	−0.003 (−0.02, 0.01)	0.56 (0.18, 0.95) *	0.83 (0.26, 1.39) *
rs10830962 ^b^	G/C	0.42	0.03 (−0.02, 0.09)	−0.003 (−0.02, 0.01)	0.57 (0.18, 0.96) *	0.83 (0.21, 1.46) *
rs4753426 ^a^	C/T	0.49	0.01 (−0.05, 0.07)	−0.01 (−0.04, 0.02)	0.30 (−0.19, 0.80)	0.42 (−0.01, 0.85) ‡
rs4753426 ^b^	C/T	0.49	0.01 (−0.05, 0.07)	−0.01 (−0.04, 0.02)	0.31 (−0.18, 0.80)	0.42 (0.01, 0.83) *
rs8192552 ^a^	G/A	0.93	−0.001 (−0.06, 0.05)	−0.02 (−0.05, 0.02)	0.31 (−0.52, 1.15)	0.28 (−0.41, 0.98)
rs8192552 ^b^	G/A	0.93	−0.001 (−0.05, 0.05)	−0.02 (−0.05, 0.02)	0.31 (−0.52, 1.14)	0.28 (−0.45, 1.01)
rs10830963 ^a^	G/C	0.29	0.04 (−0.03, 0.10)	−0.004 (−0.02, 0.02)	0.80 (0.16, 1.45) *	1.21 (0.62, 1.78) †
rs10830963 ^b^	G/C	0.29	0.04 (−0.03, 0.10)	−0.004 (−0.02, 0.02)	0.80 (0.14, 1.47) *	1.21 (0.59, 1.83) †
rs3781638 ^a^	T/G	0.55	0.01 (−0.06, 0.07)	−0.002 (−0.04, 0.03)	0.18 (−0.17, 0.54)	0.32 (−0.08, 0.71) ‡
rs3781638 ^b^	T/G	0.55	0.01 (−0.05, 0.07)	−0.002 (−0.04, 0.03)	0.18 (−0.17, 0.54)	0.31 (−0.06, 0.69) ‡
*MTNR1B* score ^a^			0.01 (−0.01, 0.02)	−0.001 (−0.01, 0.004)	0.15 (0.01, 0.30) *	0.23 (0.10, 0.36) *
*MTNR1B* score ^b^			0.01 (−0.01, 0.02)	−0.001 (−0.01, 0.004)	0.15 (0.01, 0.30) *	0.23 (0.10, 0.37) *
*MTNR1B* score >6 vs. ≤6 § ^a^			0.07 (−0.01, 0.15) ‡	0.01 (−0.01, 0.03)	0.87 (0.38, 1.36) †	1.25 (0.70, 1.80) †
*MTNR1B* score >6 vs. ≤6 § ^b^			0.07 (−0.01, 0.15) ‡	0.01 (−0.02, 0.04)	0.87 (0.38, 1.37) †	1.25 (0.68, 1.83) †
§ + Short Form-36 mental health || ^a^			0.07 (−0.01, 0.15) ‡	0.02 (−0.02, 0.06)	0.82 (0.36, 1.28) †	1.11 (0.76, 1.46) †
§ + Short Form-36 mental health || ^b^			0.07 (−0.01, 0.15) ‡	0.02 (−0.02, 0.06)	0.81 (0.36, 1.27) †	1.12 (0.74, 1.49) †

^a^ Adjusted model without BMI; ^b^ Adjusted model with BMI; § *MTNR1B* score > 6 vs. ≤6 model; * *p* < 0.05; † *p* < 0.001; ‡ *p* < 0.1. || N (all) = 1865; N (no diabetes) = 1711; N (diabetes) = 152; N (diabetes on medication) = 99.

**Table 4:** Sensitivity analyses on the association between night-time road traffic noise and change in HbA1c, in non-movers.

Model	Categories	All N = 2142	No Diabetes N = 1960	Diabetes N = 179	Diabetes on Medication N = 117
		β (95% CI)	β (95% CI)	β (95% CI)	β (95% CI)
Main model corrected for potential selection bias	NA	0.02 (0.003, 0.04) *	0.01 (0, 0.03) *	0.03 (−0.14, 0.19)	0.10 (−0.09, 0.29)
Complete case analyses: Adjusted model	NA	0.02 (−0.002, 0.05) †	0.02 (0, 0.04) *	0.03 (−0.21, 0.28)	0.05 (−0.28, 0.37)
Complete case analyses: Stratification by *MTNR1B* score	≤6	−0.01 (−0.04, 0.03)	0.01 (−0.01, 0.04)	−0.34 (−0.64, −0.04) *	−0.40 (−0.76, −0.05) *
	>6	0.06 (0.02, 0.09) *	0.03 (0.001, 0.05) *	0.49 (0.16, 0.82) *	0.81 (0.37, 1.26) ‡
	*P-value of interaction*	*<0.01*	*0.48*	*<0.001*	*<0.001*
Main model excluding participants whose bedrooms did not face the street: Stratification by *MTNR1B* score	≤6	0.01 (−0.03, 0.04)	0.02 (−0.01, 0.05)	−0.19 (−0.39, −0.003) *	−0.02 (−0.38, 0.34)
	>6	0.07 (0.01, 0.13) *	0.03 (−0.001, 0.06)	0.49 (0.08, 0.90) *	0.85 (0.23, 1.47) ‡
	*P-value of interaction*	*0.14*	*0.42*	*<0.001*	*<0.001*
Main model excluding asthma cases: Stratification by *MTNR1B* score	≤6	−0.01 (−0.03, 0.02)	0.004 (−0.02, 0.03)	−0.26 (−0.48, −0.03) *	−0.30 (−0.53, −0.07) *
	>6	0.06 (−0.003, 0.11) *	0.02 (−0.004, 0.05) *	0.50 (0.07, 0.92) *	0.98 (0.35, 1.61) *
	*P-value of interaction*	*0.07*	*0.39*	*0.001*	*<0.001*
Main model: Stratification by self-reported insufficient sleep	No	−0.002 (−0.03, 0.02)	−0.001 (−0.02, 0.02)	−0.08 (−0.28, 0.15)	−0.11 (−0.36, 0.14)
	Yes	0.07 (0.02, 0.14) *	0.05 (0.004, 0.09) *	0.28 (−0.13, 0.69)	0.67 (0.11, 1.23) *
	*P-value of interaction*	*0.092*	*0.059*	*0.2*	*0.029*

* *p* < 0.05; † *p* < 0.1; ‡ *p* < 0.001.

## Supplementary Materials

The following are available online at www.mdpi.com/1660-4601/14/12/1492/s1, Table S1: Characteristics of common *MTNR1B* variants included in the study; Table S2: Characteristics of study participants included and excluded in the study; Table S3: Interaction between *MTNR1B* variants (single/score) and night-time road traffic noise (10 dB difference) on change in HbA1c, in all participants; Table S4: Association between *MTNR1B* variants and measures of HbA1c (%); Table S5: Interaction between *MTNR1B* genetic risk score and 10 dB difference in night-time road, railway and aircraft noise, on change in HbA1c; Table S6: Characteristics of included participants stratified by moving status; Figure S1: Selection of participants for the present study; Figure S2: Linkage disequilibrium matrix for seven common *MTNR1B* variants; Figure S3: Distribution of night-time road traffic, aircraft and railway noise in the included participants.
