# Supplementary material for: Exposure to Night-Time Traffic Noise, Melatonin-Regulating Gene Variants and Change in Glycemia in Adults
Source: Int J Environ Res Public Health. 2017 Dec 1;14(12):1492. doi: 10.3390/ijerph14121492 (PMC5750910; doi:10.3390/ijerph14121492)
Supplement: Supplementary file 1 [file ijerph-14-01492-s001.pdf]

SUPPLEMENTARY MATERIAL

Table S1. Characteristics of common *MTNR1B* variants included in the study.

SNP	Risk/Alternate Allele	Risk Allele Frequency in SAPALDIA	Published Risk Allele Frequency [1]	Risk Allele Frequency from 1000 Genomes' Central European Population [2]	*P*-value of Hardy–Weinberg Equilibrium Test in SAPALDIA
rs1387153	T/C	0.30	0.30 [3]	0.28	0.49
rs10830962	G/C	0.42	0.40 [4]	0.40	0.40
rs4753426	C/T	0.49	0.50 [4]	0.51	0.69
rs8192552	G/A	0.93	0.90 [5]	0.94	0.36
rs10830963	G/C	0.29	0.30 [4,6–14]	0.29	0.21
rs3781638	T/G	0.55	0.55 [4]	0.56	0.71

Table S2. Characteristics of study participants included and excluded in the study.

Categorical Variables	Included	Excluded	P-value
	(%)	N (%)	Chi² test
Females at SAP2	48	4697 (55)	<0.001
Formal education at SAP2: ≤9 years	4	557 (12)	<0.001
10–13 years	64	3108 (66)	x
>13 years	32	1026 (22)	x
Ever-smoker at SAP2	55	4684 (60)	<0.001
Exposure to passive smoke at SAP2	23	4290 (27)	<0.001
Alcohol consumption >once/day at SAP2	40	3212 (41)	0.75
Sleep insufficiency at SAP2	28	3216 (28)	0.76
Diabetes status at SAP2	3	4071 (5)	<0.001
Diabetes status at SAP3	7	2499 (9)	0.05
Diabetes medication at SAP2	3	2458 (5)	<0.001
Diabetes medication at SAP3	5	1821 (6)	0.07
Change of residence (movers) between SAP2 and SAP3	36	2496 (38)	0.14
Continuous variables (units)	Mean (SD)	N (Mean (SD))	*t*-test
Age at SAP2 (years)	51 (11)	4697 (53 (12))	<0.001
Body mass index at SAP2 (kg/m²)	25.6 (4)	3248 (26.2 (5))	<0.001
Neighborhood socio-economic index at SAP2 (%)	64 (10)	4640 (63 (11))	<0.001
SF-36 mental health score at SAP2 (%) [b]	76 (15)	2638 (74 (16))	<0.001
Lnight, road at SAP2 (dB)	45.8 (8)	4640 (46.7 (8))	<0.001
Lnight, railway at SAP2 (dB)	28.3 (10)	4640 (28.5 (10))	0.47
Lnight, aircraft at SAP2 (dB)	22.7 (6)	4640 (23.5 (6))	<0.001
Nitrogen dioxide at SAP2 (µg/m³)	22.4 (10)	4599 (24.1 (10))	<0.001
Green space within 2 km radius at SAP2 (km²)	0.3 (0.4)	4640 (0.4 (0.4))	<0.001
MTNR1B variants at SAP2: rs1387153, T allele	0.6 (0.6)	974 (0.6 (0.7))	0.57
rs10830962, G allele	0.8 (0.7)	974 (0.8 (0.7))	0.99
rs4753426, C allele	1.0 (0.7)	974 (1.0 (0.7))	0.70
rs8192552, G allele	1.8 (0.4)	974 (1.9 (0.3))	0.01
rs10830963, G allele	0.6 (0.6)	974 (0.6 (0.6))	0.99
rs37816938, G allele	1.1 (0.7)	974 (1.0 (0.7))	0.05
MTNR1B genetic risk score at SAP2	6 (3)	974 (6 (3))	0.93
Glycosylated hemoglobin, HbA1c at SAP2 (%)	5.2 (0.4)	2844 (5.3 (0.5))	<0.001
HbA1c at SAP3 (%)	5.3 (0.5)	930 (5.2 (0.4))	0.01
Change in HbA1c between SAP2 and SAP3 (%): *All*	0.04 (0.4)	437 (0.02 (0.3))	0.35
Non-diabetic participants	0.03 (0.3)	410 (0.01 (0.3))	0.33
Diabetic participants	0.17 (1.1)	25 (0.14 (0.7))	0.88
Diabetic participants on medication	0.12 (1.2)	15 (0.06 (0.8))	0.84

[a] N(included) = 2933

The presented study included 3350 participants, with summary measures presented in the second column. "Excluded" represents participants who took part in the baseline SAPALDIA study in 1991 (SAP1) but did not participate in the present analyses due to non-participation in the follow-up surveys or missing data. SAP2 and SAP3: First and second follow-up surveys of the Swiss Cohort Study on Air Pollution and Lung and Heart Diseases in Adults in 2002 and 2010/11

respectively. Lnight represents night-time (23–07 hours) noise levels assigned to the SAPALDIA participants based on the most exposed façade of their residential floors.

Table S3. Interaction between *MTNR1B* variants (single/score) and night-time road traffic noise (10dB difference) on change in HbA1c, in all participants.

***MTNR1B* Variables**	**Risk/Other Allele**	**RAF**	**All N = 3350**	**No diabetes N = 3098**	**Diabetes N = 251**	**Diabetes on Medication N = 168**
			**β Interaction Term (95% CI)**	**β Interaction Term (95% CI)**	**β Interaction Term (95% CI)**	**β Interaction Term (95% CI)**
rs1387153	T/C	0.30	0.01 (−0.04, 0.06)	0.003 (−0.01, 0.02)	0.20 (−0.27, 0.66)	0.27 (−0.26, 0.81)
rs10830962	G/C	0.42	0.01 (−0.04, 0.06)	−0.003 (−0.02, 0.01)	0.21 (−0.47, 0.06)	0.23 (−0.32, 0.77)
rs4753426	C/T	0.49	−0.002 (−0.05, 0.04)	−0.005 (−0.03, 0.02)	0.10 (−0.27, 0.47)	0.14 (−0.21, 0.48)
rs8192552	G/A	0.93	−0.004 (−0.04, 0.04)	−0.01 (−0.05, 0.02)	0.23 (−0.27, 0.75)	0.26 (−0.33, 0.84)
rs10830963	G/C	0.29	0.01 (−0.05, 0.06)	−0.002 (−0.02, 0.01)	0.27 (−0.28, 0.83)	0.46 (−0.23, 1.15)
rs3781638	T/G	0.55	−0.003 (−0.05, 0.04)	−0.0002 (−0.02, 0.02)	0.01 (−0.36, 0.38)	0.06 (−0.32, 0.45)
MTNR1B score			0.001 (−0.01, 0.01)	−0.001 (−0.01, 0.04)	0.04 (−0.08, 0.16)	0.07 (−0.07, 0.20)
MTNR1B score > 6 vs. ≤ 6§			0.03 (−0.04, 0.09)	0.01 (−0.01, 0.03)	0.38 (−0.20, 0.97)	0.50 (−0.26, 1.27)
§ + SF-36 mental health||			0.04 (−0.02, 0.10)	0.01 (−0.02, 0.04)	0.49 (−0.05, 1.02)	0.72 (0.12, 1.32)

|| N(all)=1865; N (no diabetes) = 1711; N(diabetes)=152; N (diabetes on medication) = 99.

Interaction terms included night-time road traffic noise and *MTNR1B* variants/score. *MTNR1B* score represents the sum of the risk alleles across six included variants. Positive sign of beta coefficient means increase in HbA1c per 10dB change in night-time road traffic noise and per risk allele. All models were adjusted for age, sex, education, neighborhood socio-economic index, smoking status, passive smoking, alcohol consumption, body mass index, green space within a 2km residential buffer, residential levels of nitrogen dioxide, night-time railway, aircraft noise and their truncation indicators. All models included random intercepts at the level of the study areas, and were corrected for potential selection bias by applying the probability of participation in present analyses as weights derived from a logistic regression with predictors from the baseline study in 1991. SF-36: Short Form 36-item survey.

Table S4. Association between *MTNR1B* variants and measures of HbA1c (%).

***MTNR1B* Variants**	**Repeated Measures of HbA1c at SAP2 and SAP3**				**Change in HbA1c Between SAP2 and SAP3**			
	**β (95% CI)**				**β (95% CI)**			
	**All N = 6641**	**No Diabetes N = 6298**	**Diabetes N = 338**	**Diabetes on Medication N = 214**	**All N = 3350**	**No Diabetes N = 3094**	**Diabetes N = 251**	**Diabetes on Medication N = 168**
rs1387153	0.01 (−0.001, 0.03)	0.02 (0.001, 0.04) *	−0.11 (−0.24, 0.01)	−0.06 (−0.29, 0.18)	−0.01 (−0.02, 0.01)	0.01 (−0.01, 0.02)	−0.21 (−0.37, −0.06) *	−0.23 (−0.48, 0.01) *
rs10830962	0.002 (−0.01, 0.01)	0.01 (0.001, 0.02) *	−0.17 (−0.35, 0.002)	−0.08 (−0.31, 0.15)	−0.01 (−0.02, 0.002)	−0.001 (−0.01, 0.01)	−0.11 (−0.32, 0.11)	−0.02 (−0.32, 0.28)
rs4753426	0.02 (−0.004, 0.04)	0.02 (0.002, 0.03) *	−0.01 (−0.11, 0.09)	0.05 (−0.20, 0.30)	0.003 (−0.01, 0.02)	0.001 (−0.01, 0.02)	−0.01 (−0.25, 0.23)	0.02 (−0.25, 0.29)
rs8192552	0.03 (−0.01, 0.07)	0.03 (−0.005, 0.06)	0.12 (−0.19, 0.44)	0.10 (−0.39, 0.59)	0.02 (−0.03, 0.06)	0.01 (−0.03, 0.05)	0.07 (−0.32, 0.45)	0.06 (−0.49, 0.60)
rs10830963	0.01 (−0.004, 0.02)	0.02 (0.0005, 0.04) *	−0.19 (−0.36, −0.02) *	−0.05 (−0.31, 0.21)	−0.01 (−0.03, 0.0003)	0.01 (−0.01, 0.02)	−0.27(−0.49, −0.05) *	−0.21 (−0.53, 0.12)
rs3781638	0.005 (−0.01, 0.02)	0.01 (−0.001, 0.02)	−0.07 (−0.22, 0.09)	−0.01 (−0.28, 0.26)	0.001 (−0.02, 0.02)	0.003 (−0.01, 0.02)	−0.02 (−0.29, 0.26)	0.01 (−0.29, 0.31)
MTNR1B score	0.003 (−0.0002, 0.01)	0.005 (0.001, 0.01) *	−0.03 (−0.06, 0.004)	−0.005 (−0.07, 0.06)	−0.001 (−0.004, 0.002)	0.001 (−0.002, 0.005)	−0.03 (−0.08, 0.02)	−0.02 (−0.09, 0.06)
MTNR1B score > 6 vs. ≤ 6	0.01 (−0.01, 0.04)	0.03 (0.003, 0.05) *	−0.32 (−0.53, −0.11) *	−0.21 (−0.52, 0.10)	−0.01 (−0.01, 0.002)	0.003 (−0.01, 0.02)	−0.10 (−0.31, 0.11)	−0.01 (−0.32, 0.29)

* P < 0.05.

SAP2 and SAP3: First and second follow-up survey of the Swiss Cohort Study on Air Pollution and Lung and Heart Diseases in Adults in 2002 and 2011 respectively. Change in HbA1c defined as the absolute difference between HbA1c at SAP3 and HbA1c at SAP2. *MTNR1B* score represents the sum of the risk alleles across six included *MTNR1B* variants. Positive sign means increase in HbA1c per risk allele. Models adjusted for age, sex, education, neighborhood socio-economic index, smoking status, passive smoking and alcohol consumption. Repeated measures model additionally adjusted for survey. All models were corrected for potential selection bias by applying the probability of participation in present analyses as weights derived from a logistic regression with predictors from the baseline study in 1991. Random intercepts were applied at the levels of the participants (repeated measures models) and study areas (change in HbA1c models).

Table S5. Interaction between *MTNR1B* genetic risk score and 10dB difference in night-time road, railway and aircraft noise, on change in HbA1c.

All Participants	*MTNR1B* Variable	All N = 3350	No Diabetes N = 3098	Diabetes N = 251	Diabetes on Medication N = 168
		β Interaction Term (95% CI)	β Interaction Term (95% CI)	β Interaction Term (95% CI)	β Interaction Term (95% CI)
Lnight road	*MTNR1B* score	0.001 (−0.01, 0.01)	−0.001 (−0.01, 0.04)	0.04 (−0.08, 0.16)	0.07 (−0.07, 0.20)
	MTNR1B score > 6 vs. ≤6	0.03 (−0.04, 0.09)	0.01 (−0.01, 0.03)	0.38 (−0.20, 0.97)	0.50 (−0.26, 1.27)
Lnight railway	*MTNR1B* score	0.004 (−0.004, 0.01)	0.003 (−0.002, 0.01)	0.04 (−0.03, 0.11)	0.05 (−0.02, 0.13)
	MTNR1B score > 6 vs. ≤6	0.02 (−0.01, 0.05)	0.01 (−0.01, 0.03)	0.16 (−0.05, 0.37)	0.15 (−0.06, 0.36)
Lnight aircraft	*MTNR1B* score	0.001 (−0.003, 0.006)	0.002 (−0.002, 0.005)	−0.02 (−0.12, 0.09)	−0.002 (−0.12, 0.11)
	MTNR1B score > 6 vs. ≤6	0.01 (−0.02, 0.03)	−0.001 (−0.02, 0.02)	−0.07 (−0.51, 0.37)	0.12 (−0.40, 0.64)
**Non-movers**	***MTNR1B* Variable**	**All N = 2142**	**No diabetes N = 1960**	**Diabetes N = 179**	**Diabetes on medication N = 117**
Lnight road	*MTNR1B* score	0.01 (−0.01, 0.02)	−0.001 (-0.01, 0.004)	0.15 (0.01, 0.30) *	0.23 (0.10, 0.37) *
	MTNR1B score > 6 vs. ≤6	0.07 (−0.01, 0.15) ‡	0.01 (-0.02, 0.04)	0.87 (0.38, 1.37) *	1.25 (0.68, 1.83) †
Lnight railway	*MTNR1B* score	0.003 (−0.01, 0.01)	0.003 (-0.002, 0.01)	0.03 (−0.06, 0.11)	0.03 (−0.07, 0.14)
	MTNR1B score > 6 vs. ≤6	0.02 (−0.03, 0.06)	0.01 (-0.01, 0.04)	0.10 (−0.17, 0.38)	0.14 (−0.22, 0.49)
Lnight aircraft	*MTNR1B* score	−0.001 (−0.01, 0.005)	0.003 (-0.001, 0.01)	−0.07 (−0.22, 0.09)	−0.03 (−0.17, 0.11)
	MTNR1B score > 6 vs. ≤6	−0.01 (−0.05, 0.02)	0.01 (-0.01, 0.03)	−0.37 (−0.94, 0.20)	0.01 (−0.53, 0.55)

‡ P < 0.1; * P < 0.05; † P < 0.001.

.

All results are presented as increase or decrease in mean change in HbA1c per 10 units of exposure. *MTNR1B* score represents the sum of the risk alleles across six included variants. All models were multi-exposure models including night-time road traffic noise, aircraft noise, railway noise and nitrogen dioxide, adjusted for age, sex, education, neighborhood socio-economic index, smoking status, passive smoking, alcohol consumption, body mass index, and green space within 2km residential buffer. All models included random intercepts at the level of the study areas, and were corrected for potential selection bias by applying the probability of participation in present analyses as weights derived from a logistic regression with predictors from the baseline study in 1991.

Table S6. Characteristics of included participants stratified by moving status.

**Categorical Variables**	**Non-movers**	**Movers**	**P-value**
	**2142 (%)**	**1192 (%)**	**Chi² test**
Females at SAP2	49	46	0.13
Formal education at SAP2: ≤9 years	5	3	0.12
10–13 years	64	65	
>13 years	31	32	
Ever-smoker at SAP2	55	56	0.01
Exposure to passive smoke at SAP2	21	28	<0.001
Alcohol consumption >once/day at SAP2	41	39	0.19
Sleep insufficiency at SAP2 [a]	27	29	0.12
Diabetes status at SAP2	3	3	0.39
Diabetes status at SAP3	8	6	0.02
Diabetes medication at SAP2	3	2	0.71
Diabetes medication at SAP3	6	4	0.07
Continuous variables (units)	Mean (SD)	Mean (SD)	T-test
Age at SAP2 (years)	53 (10)	48 (11)	<0.001
Body mass index at SAP2 (kg/m^2)	25.7 (4)	25.5 (5)	0.15
Neighborhood socio-economic index at SAP2 (%)	65 (10)	63 (10)	<0.001
SF-36 mental health score at SAP2 (%) [b]	77 (14)	74 (16)	<0.001
Lnight, road at SAP2 (dB)	45.6 (8)	46.4 (8)	0.004
Lnight, railway at SAP2 (dB)	28.6 (10)	27.8 (11)	0.04
Lnight, aircraft at SAP2 (dB)	22.9 (6)	22.3 (5)	0.003
Nitrogen dioxide at SAP2 (μg/m^3)	22.8 (9)	21.7 (10)	0.003
Green space within 2 km radius at SAP2 (km^2)	0.3 (0.4)	0.3 (0.4)	0.76
MTNR1B variants at SAP2: rs1387153, T allele	0.6 (0.6)	0.6 (0.6)	0.23
rs10830962, G allele	0.8 (0.7)	0.8 (0.7)	0.57
rs4753426, C allele	1.0 (0.7)	1.0 (0.7)	0.45
rs8192552, G allele	1.8 (0.4)	1.9 (0.4)	0.48
rs10830963, G allele	0.6 (0.6)	0.5 (0.6)	0.16
rs37816938, G allele	1.1 (0.7)	1.1 (0.7)	0.33
MTNR1B genetic risk score at SAP2	6 (3)	6 (3)	0.30
Glycosylated hemoglobin, HbA1c at SAP2 (%)	5.3 (0.5)	5.2 (0.4)	<0.001
HbA1c at SAP3 (%)	5.3 (0.5)	5.2 (0.5)	0.03
Change in HbA1c between SAP2 and SAP3 (%): *All*	0.03 (0.4)	0.06 (0.4)	0.04
Non-diabetic participants	0.02 (0.3)	0.05 (0.3)	0.01
Diabetic participants	0.14 (1.1)	0.27 (1.3)	0.40
Diabetic participants on medication	0.10 (1.2)	0.30 (1.3)	0.24

[a] N(non-movers) = 1870, N(movers) = 1047.

The presented study included 3350 participants, with summary measures presented in the second column. "Excluded" represents participants who took part in the baseline SAPALDIA study in 1991 (SAP1) but did not participate in the present analyses due to non-participation in the follow-up surveys or missing data. SAP2 and SAP3: First and second follow-up surveys of the Swiss Cohort Study on Air Pollution and Lung and Heart Diseases in Adults in 2002 and 2010/11

respectively. Lnight represents night-time (23–07 hours) noise levels assigned to the SAPALDIA participants based on the most exposed façade of their residential floors.

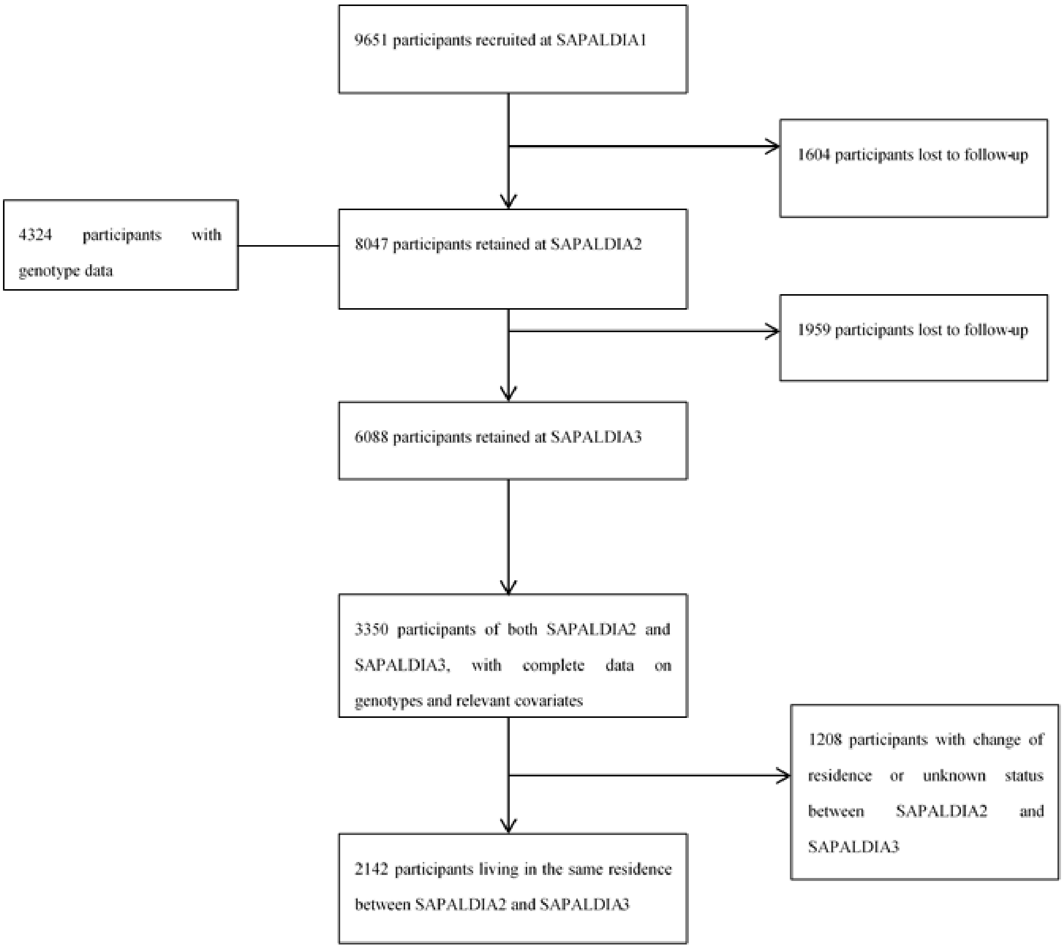

Figure S1. Selection of participants for the present study.

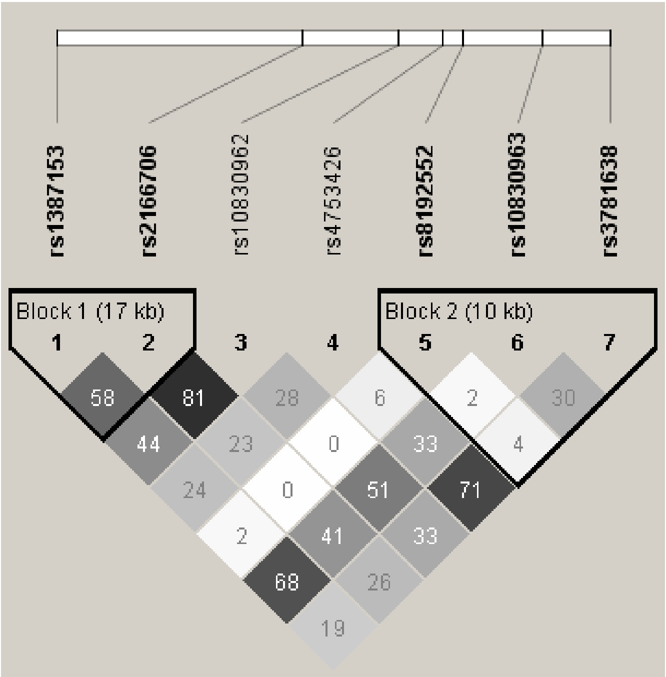

Figure S2. Linkage disequilibrium matrix for seven common *MTNR1B* variants.

MTNR1B genetic locus showing the locations and the linkage disequilibrium of seven common functional variants within this region based on the 1000 Genomes project. Vertical lines on chromosome represent the locations of the variants. The color scheme and numbers within the diamonds are based on the R^2 values of linkage disequilibrium between the selected variants.

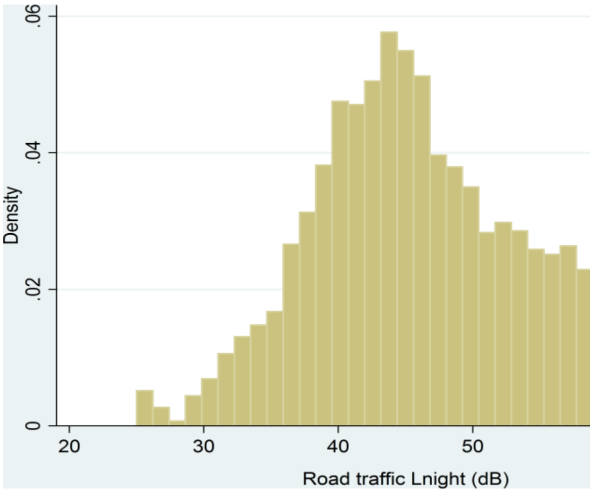

(**a**)

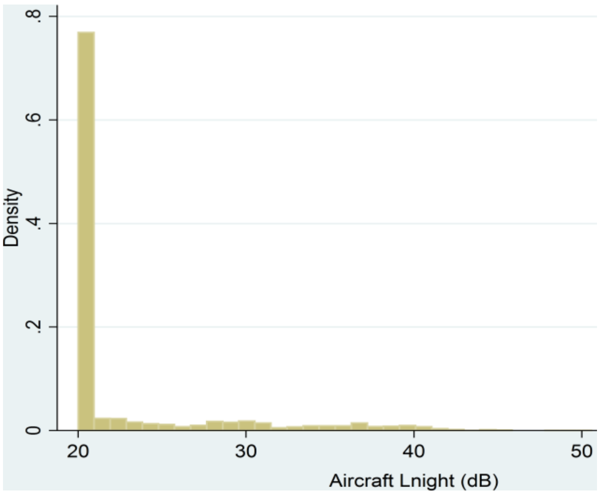

(**b**)

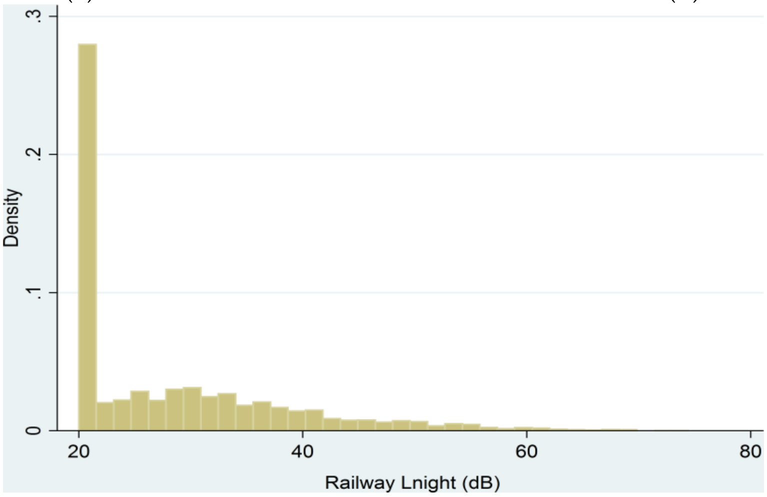

(**c**)

Figure S3. Distribution of night-time road traffic (**a**), aircraft (b) and railway noise (**c**) in the included participants.

All noise estimates were calculated for 2001, between 2300-0700 hours, using validated Swiss noise models and assigned to participants based on the most exposed façade of their residential floors. Road traffic noise was modelled using SonBASE emission and STL-86 propagation models. Aircraft noise was modelled using FLULA2, and railway noise was modelled using SonRAIL emission and SEMIBEL propagation models.
